# Comparative analysis of fermented sausages from purebred and crossbred Bamei pig: physicochemical properties, fatty acids, microbiota, and metabolites

**DOI:** 10.1016/j.fochx.2025.103299

**Published:** 2025-11-19

**Authors:** Sen Xiang, Youqing Wang, Jiale Zheng, Zhongfang Tan, Guofang Wu, Lei Wang, Jianbo Zhang, Xuan Luo, Xin Zhang, Haiying Wang

**Affiliations:** a College of Life Science, South-Central MinZu University, Wuhan, Hubei 430074, China; b Henan Key Laboratory of lon-Beam Green Agriculture Bioengineering, School of Agricultural Sciences, Zhengzhou University, Zhengzhou, Henan 450052, China; c Innovative Utilization Key Laboratory of Qinghai Province, Key Laboratory of Animal Genetics and Breeding on Tibetan Plateau, Academy of Animal Science and Veterinary Medicine, Qinghai University, Xining, Qinghai 810016, China

**Keywords:** Pure Bamei pork, Crossbred pork, Fermented sausages, Physicochemical properties, Flavor compounds, Metabolomic

## Abstract

The Bamei pig, an indigenous Chinese breed renowned for meat quality, remain poorly characterized for fermentation processing performance. The aim of this study was to compare the effects of Pure Bamei Pigs and its binary, ternary crossbred pork on physicochemical properties, microbiota, and flavor of fermented sausages. Ternary sausages exhibited the best sensory scores, the lowest water activity (A_w_) and the richest profiles of olefins and esters. Metabolomics analyses showed that the most significant metabolic differences among the three groups of sausages centered on lipid metabolism. The polyunsaturated fatty acid contents, especially linoleic acid, were significantly higher in crossbred pork sausages than in Pure Bamei sausages. Microbiota analysis identified *Lactobacillus* and *Leuconostoc* were the core functional genera, was positively related to with fatty acid composition, ester alcohols, and terpenoids. Overall, ternary-cross Bamei pork is recommended as an optimal raw material for fermented Chinese sausages.

## Highlights

• Sausages made from ternary pork have better sensory characteristics.
• Ternary pork sausages possess superior in high linoleic acid content.
• The VOCs of Ternary pork Sausage are rich in olefins and high content of esters.
• *Lactobacillus* and *Leuconostoc* were the core functional genera.
• The most significant metabolic differences centered on lipid metabolism.

## 1 Introduction

The Bamei pig was recognized as a significant indigenous genetic resource in China, distinguished by its strong adaptability, stress resistance, reproductive capacity, and stable genetic traits (Chen et al., 2020). Plateau ecotypes subjected to long-term natural and artificial selection produce pork with their high fat deposition, excellent meat quality, and unique flavor. However, their slow growth, poor rear development and thick skin severely constrain commercial use (Li et al., 2013).

In order to fully develop this excellent local pig breed resource, crossbreeding with introduced breeds is a common practice. The Pure Bamei pig was first crossed with the Bakosi pig to produce a binary cross, and then crossed with the Duroc pig to produce a ternary cross. These crossbred pigs have shown great economic value due to faster growth rates, higher feed conversion rates, and higher meat production rates (Zhou et al., 2016). However, studies have shown significant differences in key meat quality parameters, including water loss rate, shear force, marbling score, fatty acid composition, and volatile components between Pure Bamei and their crossbred porks (Chen & Sui, 2017). These differences suggest that genetic background impacts meat physicochemical properties.

Fermentation of seasoned raw meat into Chinese sausages offers an effective valorization route for indigenous pork, yielding shelf-stable products with distinctive color, texture and flavor (Kumar et al., 2015). It is generally recognized that microorganisms, especially lactic acid bacteria play a crucial role in suppressing pathogens and ensuring safety, stability, and distinctive sensory traits (Bedia et al., 2011). However, increasing evidence suggests that the intrinsic properties of the raw material are also critical in determining the quality of traditional fermented meat products. Schalkwyk et al. analyzed salamis made from different species of meat (springbok, gemsbok, kudu, and zebra) and found differences in sensory attributes (van Schalkwyk et al., 2011). Settanni found differences in branched-chain fatty acids and rumenic acid in salamis made from beef, horse, wild boar, and pork (Settanni et al., 2020). Škrlep reported that traditionally prepared pork sausages had advantages in consumer acceptability, texture, and diversity of volatile flavor compounds compared to organic pork (Škrlep et al., 2019). Panea & Ripoll found that dietary plant extracts, including garlic and carvacrol, can shape the quality of raw meat and consequently the physicochemical properties and consumer perception of fermented sausages (Panea & Ripoll, 2020). These studies have demonstrated that the raw meat characteristics, which are determined by genetic background, feeding regimens, and postmortem biochemical reactions, significantly form the quality of fermented meat products. Crossbreeding alters these variables, but systematic comparisons of fermented sausages made from purebred and crossbred pork remain scarce.

With this in mind, pork of Pure Bamei, binary crossbred and ternary crossbred were processed into Chinese sausages under identical conditions. By comparing of the physicochemical properties, fatty acid composition, and volatile compounds, the impact of pig genetic background on the quality of fermented sausages was revealed. Furthermore, through analysis of microbial communities and metabolites, the mechanisms by which crossbreeding affects sausage quality were elucidated, and the pathways involved in the formation of characteristic flavor compounds were preliminarily identified. It guides raw-material choice for Bamei pork sausages and promoting the high-value utilization of local pig genetic resources. The study could enhance the comprehensive understanding of how the genetic background of pigs influences the fermentation process and quality of sausage.

## 2 Material and methods

### 2.1 Source of sausage raw materials

The Pure Bamei pork, binary crossbred pork, and ternary crossbred pork were sourced from Bamei Pig Original Breeding Base of Huzhu County, Qinghai Province, China. All experimental pigs were maintained under standardized husbandry conditions and slaughtered according to the same procedure. *Lactobacillus curvatus SQ-425* were screened from Sichuan preserved meat and sausages and are preserved at the China Center for Type Culture Collection (CCTCC NO: M 2024351). White wine, sodium nitrite and sucrose were purchased from a local market (Xining, China).

### 2.2 Preparation of fermented sausages

Three groups of sausages were prepared on the same day using the front leg of Pure Bamei pork, binary crossbred pork and ternary crossbred pork, respectively. Each group consisted of three replicate batches, with each batch sourced from a different carcass. *L. curvatus SQ-425* were incubated in the DeMan, Rogosa and Sharpe (MRS) broth at 37 °C. The cell pellet was acquired by centrifuge at 5000 rpm for 10 min and preserved at 4 °C for use.

**Fig. 1:**
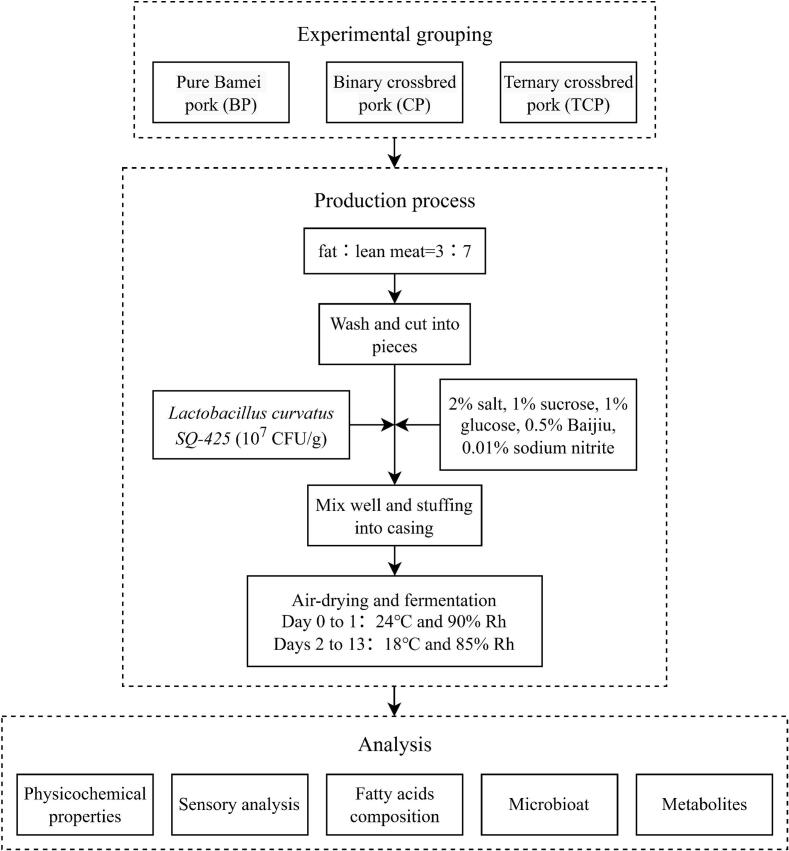
Experimental Design and Workflow Overview.

The sausages were made with a lean meat to fat ratio of 7:3, seasoned with 2 % salt, 1 % sucrose, 1 % glucose, 0.5 % white wine, and an additional 0.01 % sodium nitrite. *L. curvatus* SQ-425 was inoculated at a concentration of 10^7^ CFU per gram of meat in batches, stirred well and stuffed into pig natural casings. Fermentation was conducted at 24 °C and 90 % relative humidity for 24 h, followed at 8 °C and 65 % relative humidity for 27 days. All experimental groups were treated under strictly the same fermentation conditions. Samples of fermented sausages were collected for analysis at days 0, 1, 4, 8, 12, 16, 20, 24, and 28 of the fermentation processes. These samples were then stored at −20 °C. A flowchart illustrating production techniques and experimental procedures is presented in graphical form as shown in Fig. 1. The fermentation process of the sausages and the samples are shown in Fig. S1.

### 2.3 Determination of pH and A_w_ (water activity)

2 g of sausage samples were homogenized with 20 mL of distilled water, and the pH of the homogenate was measured with a pH meter (Sartorius PB-10, Sartorius Scientific Instruments Beijing Co., Ltd., China). The minced sausage samples were placed in a calibrated water activity meter (AW-1 Smart Water Activity Meter, Wuxi Bibo Electronic Equipment Factory, China) to measure the water activity of the sausage samples at 25 °C.

### 2.4 Color difference and texture analysis

A calibrated colorimeter (Spectrocolorimeter PS2040, Sanenshi Technology Co., Ltd., Shenzhen, China) was used to measure the fresh cross-section of the sausages. Sausages were cut into cylinders approximately 1.5 cm in height and texture was measured using a texture analyzer (CT3 Texture Analyzer, Brookfield, USA) with a TA3/100 probe and TA-SBA clamp, applying a non-destructive deformation at a speed of 2.00 mm/s to 30 % deformation, with a load limit of 10,000 g, to obtain indices such as hardness, elasticity, and chewiness.

### 2.5 Sensory evaluation

The sensory evaluation was conducted in a food laboratory. The sensory evaluation panel consisted of 20 members, including 10 females and 10 males, aged between 25 and 45 years, all of whom had backgrounds in disciplines related to food science. All panel members received professional training in sensory evaluation. The sensory evaluation criteria focused on the assessment of the color, texture, taste, and flavor of sausages. The evaluation criteria and their respective scores are shown in Table S2 (Armenteros et al., 2009).

Ethical permission to conduct this sensory study was not required by our institution and country. However, we confirm that all participants were treated in accordance with ethical research standards. Informed consent was obtained from all participants prior to their involvement in the study, and their rights and privacy were protected throughout the research process. Participants were provided with detailed information about the study's purpose and procedures, and their verbal consent was obtained before participation. All data were anonymized to protect participant identities, and participants were informed of their right to withdraw at any stage without consequence.

### 2.6 Determination of volatile basic nitrogen and histamine

The determination of volatile basic nitrogen (TVB-N) and histamine in the sausages was conducted using the Kjeldahl method with a K-360 fully automatic Kjeldahl nitrogen analyzer (BUCHI Labortechnik AG, Switzerland), following the study of Chen (Cheng et al., 2024). Additionally, the histamine content was assessed using high-performance liquid chromatography (HPLC system G7129A, Agilent Technologies Inc., USA), adhering to the detailed protocols provided in the research of Vasconcelos (Vasconcelos et al., 2021).

### 2.7 Determination of fatty acids composition

The fatty acid composition was measured according to the method of Li et al. (Li et al., 2023). Crude fat was extracted from sausage samples using a fat extractor (SZF—06C, Zhejiang Top Instrument Co., Ltd., China), and an internal standard (heptadecanoic acid) was added to the crude fat, and then the fatty acids were methylated as well as dewatered for determination by chromatography-mass spectrometry (GC/MS, ISQ7000, TRACE1300, Thermo Fisher Scientific, USA). A DB-WAX capillary column (30.0 m × 0.25 mm, 0.25 μm, Agilent, US) was used. The determination conditions were as follows: the pressure was set at 7.069 psi. 1 μL of the methylated solution was injected in split mode with a split ratio of 10:1. The temperature of the injection port and the detector was 230 °C, and the initial temperature of the column chamber was set at 170 °C and kept for 1 min, and the temperature was programmed to increase to 230 °C at a rate of 3 °C/min and kept for 3 min.

### 2.8 Determination of volatile organic compounds

Sausage samples was ground in liquid nitrogen with the addition of standard (2,4,6-trimethylpyridine) and analyzed by GC/MS (ISQ7000, TRACE1300, Thermo Fisher Scientific, USA). A CAR/PDMS-coated SPME fiber was exposed in the headspace vial at 90 °C for 30 min. After extraction, the CAR/PDMS fiber was inserted into the inlet port and thermally desorbed at 250 °C for 5 min. According to the method of Wang et al. (Wang et al., 2022), DB-Wax column (30 m × 0.25 mm × 0.25 μm) was used for the separation of volatile compounds. The GC temperature program was as follows: held at 40 °C for 3 min, then ramped at 4 °C/min to 150 °C for 5 min, further ramped at 5 °C/min to 200 °C, and finally ramped at 10 °C/min to 230 °C for 3 min. The ion source temperature was set at 230 °C, and the mass spectrometer scanned from 50 to 350 *m*/*z*. Volatile compounds were identified using the NIST 14 mass spectral library in combination with compound retention indices and compound mass matches greater than 80 % (RSI > 800).

### 2.9 Microbial community analysis

Total genomic DNA was extracted using the TGuide S96 Magnetic Soil/Stool DNA Kit (Tiangen Biotech (Beijing) Co., Ltd.) following the manufacturer's instructions. The V3-V4 hypervariable regions of the bacterial 16S rRNA gene were amplified with primer pairs 338F and 806R. PCR products were purified using the Omega DNA purification kit (Omega Inc., Norcross, GA, USA) and sequenced on the Illumina Novaseq 6000 platform. According to Lv’s report (Lv et al., 2023), Sequences with more than 97 % similarity were clustered into operational taxonomic units (OTUs) using USEARCH (version 10.0). Taxonomic annotation was performed using the SILVA database (release 138.1) with a 70 % confidence threshold in QIIME2 (Bolyen et al., 2020). Alpha diversity was calculated using QIIME2, and beta diversity was analyzed by principal coordinate analysis (PCoA). One-way ANOVA was used to compare bacterial abundance and diversity, and LEfSe was applied for differentially abundant taxa. Sequencing data were analyzed using the online platform BMKCloud (https://www.biocloud.net).

### 2.10 Determination of non-volatile metabolites

The LC/MS system for metabolomics analysis consists of an Ultim3000 high-performance liquid chromatography tandem Orbitrap Exploris 480 high-resolution mass spectrometer. The column used was a Waters Acquity UPLC HSS T3 column (1.8 μm, 2.1 × 100 mm). Mobile phases for positive and negative ion modes were 0.1 % formic acid aqueous solution (A) and 0.1 % formic acid acetonitrile (B). The injection volume was 1 μL. The Q Orbitrap mass spectrometer was operated in data-dependent acquisition (DDA) mode, scanning from *m*/*z* 67–1000. ESI source parameters were as follows: spray voltage 3500 V (positive) or -2500 V (negative); sheath gas flow 50 arb; auxiliary gas flow 10 arb; sweep gas flow 1 arb; ion transfer tube temperature 325 °C; vaporizer temperature 350 °C.

### 2.11 Statistical analysis

The indicators of all samples in each group was measured in triplicate. One-way ANOVA with Duncan's multiple range test and Student's *t*-test were used to assess statistical differences at *p* < 0.05 in SAS 9.2. PCA was applied to both volatile (VOMs) and non-volatile (NVOMs) metabolite profiles to visualize the overall separation among fermented sausage samples. Differential metabolites were then identified by OPLS-DA, retaining only those with FC > 1, *p* < 0.05, and VIP value (Variable Importance in Projection) > 1. Principal Component Analysis (PCA) and Partial Least Squares Discriminant Analysis (PLS-DA) were performed using SIMCA 14.1 (Umetrics, Sweden) to visualize sample differences. VIP values in the PLS-DA model indicated variable contributions to classification. Correlations between selected compounds, sensory quality, and microbial communities were calculated using Pearson correlation coefficients and visualized in R. Graphs were plotted using GraphPad Prism 9.5.0 (GraphPad Software, USA) and Origin 9.0 (OriginLab Corp, USA).

## 3 Results and discussion

### 3.1 Analysis of pH and A_w_ (water activity)

**Fig. 2:**
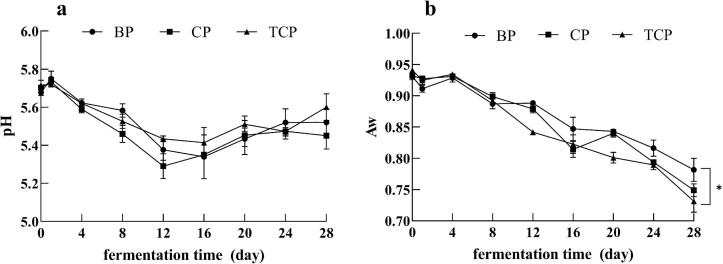
The pH (a) and A_w_ (b) contents of sausages during the fermentation. BP, Pure Bamei pork group; CP, binary crossbred pork group; TCP, ternary crossbred pork group. *means data between two groups are significantly different (*p* < 0.05), **means data between two groups are significantly different (*p* < 0.01).

As shown in Fig. 2a. The trend of pH change was similar in the three groups of sausages during fermentation. There was a rapid decline in pH values at the beginning of the fermentation (0–12 days), followed by stabilization, and the final pH of the three groups of sausages was about 5.5, with no significant differences among them (*p* > 0.05). This is similar to the process of pH change during sausage fermentation observed in the study of Wang (Wang et al., 2021). The initial decrease in pH is due to the rapid growth of lactic acid bacteria, which resulted in the substantial production of organic acids (Lv et al., 2023). The three different raw meats had no effect on the change in pH during sausage fermentation.

The A_w_ changes of the sausages are shown in Fig. 2b. The A_w_ values of the three groups of sausages continued to decrease with fermentation time and the decreasing trend was similar. At the end of fermentation, the A_w_ values of sausages made from both crossbred pork were lower than those of the Pure Bamei pork. The A_w_ value of sausages produced from Ternary crossbred pork was 0.73, which was significantly lower compared to that of Pure Bamei pork sausages, with an A_w_ of 0.78 (*p* < 0.05).

### 3.2 Analysis of color, texture, and sensory evaluation

**Fig. 3:**
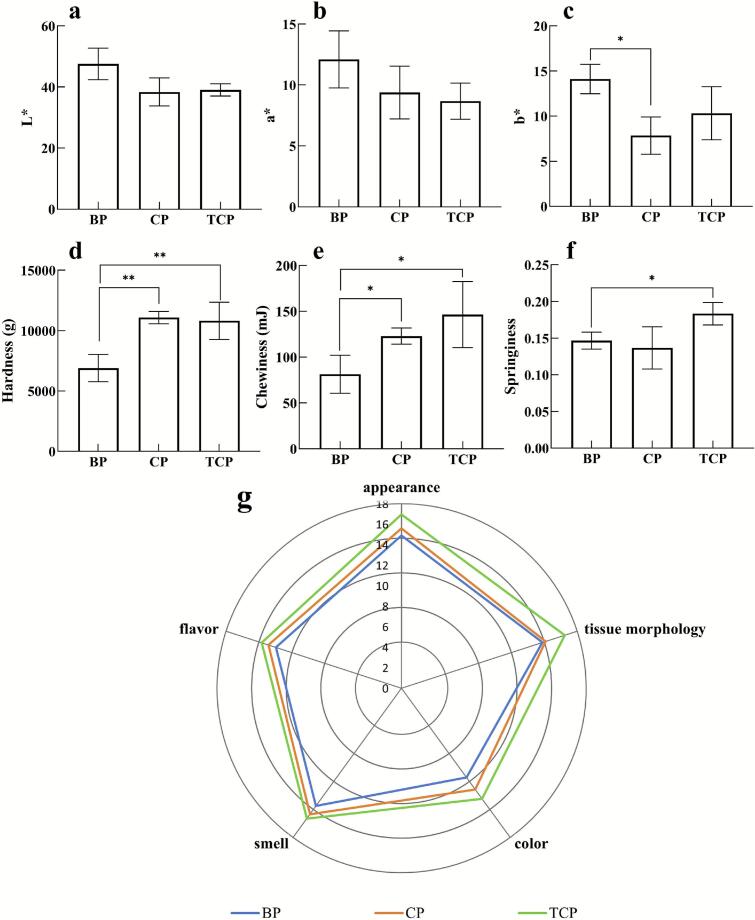
The color difference (a-c), textural properties (d-f), and sensory scores (g) of matured sausages. L*, lightness value; a*, redness value; b*, yellowness values. BP, Pure Bamei pork group; CP, binary crossbred pork group; TCP, ternary crossbred pork group. *means data between two groups are significantly different (*p* < 0.05), **means data between two groups are significantly different (*p* < 0.01).

Color and texture are one of the criteria for evaluating sausage and directly affected consumer acceptance. The color difference values are shown in Fig. 3 a -c. The redness value (a*) reflects preferences of consumers for color and is crucial to evaluating appearance quality of a meat product. The a* value for three groups of sausages were similar, with no significant differences among the groups. Compared to the lightness value (L*) of 47.54 for Pure Bamei pork sausages, the L* values of binary and ternary crossbred pork sausages significantly decreased (*p* < 0.05) at 38.37 and 39.03, respectively. The yellowness values (b*) for the three groups of fermented sausages varied significantly. The b* values of the crossbred pork sausages were significantly lower (*p* < 0.05), with the least yellowness value for the binary pork, which was 44.3 % lower compared to Pure Bamei pork. Textural properties, including hardness, elasticity, and chewiness, of fermented sausage samples at maturity were assessed (Fig. 3 d-f). Compared to Pure Bamei pork sausages, the hardness and chewiness of binary and ternary crossbred pork sausages were significantly higher (*p* < 0.05). The hardness of ternary and binary crossbred sausages was 36.1 % and 37.7 % higher than that of Pure Bamei pork sausages, respectively. Škrlep's research indicates that fermented sausages with higher hardness have relatively better acceptability (Škrlep et al., 2019). The chewiness of pork sausage was 143.1 mJ and 122.9 mJ for the ternary and binary crossbred pork, respectively, which were 1.76 and 1.51 times higher than that of Pure Bamei pork. The elasticity value of ternary crossbred pork sausages was 0.18, which was significantly higher than that of Pure Bamei pork sausages (*p* < 0.05).

Sensory evaluation is essential to estimate the acceptability of food. Sensory evaluations of the sausages were carried out and radar charts were conducted as shown in Fig. 3 g. Genetic background of pigs had a significant effect on the sensory characteristics of the fermented sausages. The scores for sausages made from Pure Bamei pork, binary crossbred and ternary crossbred pork were 67.2, 71.4, and 77.1, respectively, with significant differences in overall acceptability scores observed (*p* < 0.05). The sausages made from ternary crossbred pork, characterized by their bright red color, good chewiness, and distinct fermented aroma, scoring the highest in all five sensory evaluations.

### 3.3 Analysis of the volatile basic nitrogen (TVB-N) and histamine

**Fig. 4:**
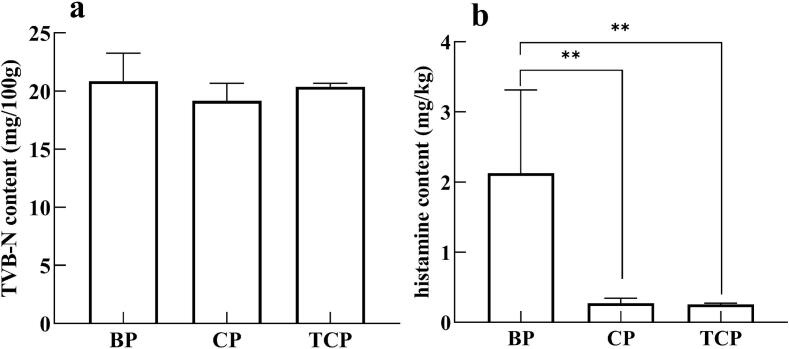
The TVB-N (a), and histamine (b) of matured sausages. TVB-N, total volatile basic nitrogen. BP, Pure Bamei pork group; CP, binary crossbred pork group; TCP, ternary crossbred pork group.*means data between two groups are significantly different (*p* < 0.05), **means data between two groups are significantly different (*p* < 0.01).

TVB-N mainly reflects the degree of protein decomposition of meat products by endogenous proteases and microorganisms, and is suggested as an important indicator of the freshness of meat products. As shown in Fig. 4a, no significant difference (*p* > 0.05) was observed in the TVB-N values at 28th day of fermentation for all groups, which were about 20 mg/100 g, similar to the value at 20th day of fermentation in the study of Fu et al. (Fu et al., 2022). This indicates that the protein degradation process during fermentation is controllable and that the use of Pure Bamei and crossbred pork did not have a significant effect on the TVB-N content.

Biogenic amines are low molecular weight nitrogen-containing organic compounds commonly found in fermentation products and can cause serious health hazards at elevated concentrations (Akan and Özdestan-Ocak, 2021). Among them, histamine is a highly toxic biogenic am2024ine that can induce adverse reactions such as headache and allergic reactions (Yu et al., 2024). As shown in Fig. 4b, the histamine content of Pure Bamei pork sausage was 2.13 mg/kg. In comparison, the histamine levels in sausages made from binary crossbred pork and ternary crossbred pork were significantly lower, at 0.27 mg/kg and 0.25 mg/kg, respectively (*p* < 0.05). This suggests that using Bamei crossbred pork as the raw material for sausage preparation can effectively reduce the histamine content in the final product. The level of histamine in food should not exceed 100 mg/kg according 91–493-EEC of the regulation of European Community (Li et al., 2019). In this study, the histamine content of all three groups were well below this threshold.

### 3.4 Analysis of fatty acids composition

The fatty acid composition of three types of raw meats and their fermented sausages was shown in Table 1. The predominant fatty acids detected in both the raw meats and the fermented sausages were palmitic acid (C16:0), stearic acid (C18:0), oleic acid (C18:1), and linoleic acid (C18:2). The monounsaturated fatty acid (MUFAs) content of all three raw meats was about 50 %, with no significant difference between groups (*p* > 0.05). The content of saturated fatty acids (SFAs) ranged from 38.10 % to 40.84 % in all groups and there was also no significant difference between the groups (*p* > 0.05). In contrast, the content of polyunsaturated fatty acids (PUFAs) differed significantly among the three groups (*p* < 0.05), with the highest PUFAs content of 12.34 % in ternary crossbred pork, 9.80 % and 11.74 % in binary crossbred pork and Pure Bamei pork, respectively.

**Table 1:** Fatty acid composition of raw meat and sausages.

Types	Pure Bamei pork (%)		Binary crossbred pork (%)		Ternary crossbred pork (%)	
	raw meat	sausage	raw meat	sausage	raw meat	sausage
C14:0	1.50 ± 0.14^A^	1.28 ± 0.01^Ba^	1.81 ± 0.10^A^	0.99 ± 0.02^Bb^	1.73 ± 0.19^A^	0.80 ± 0.08^Bc^
C16:0	25.25 ± 1.97^A^	26.97 ± 0.22^Aa^	26.25 ± 0.35^A^	23.30 ± 0.13^Bb^	25.42 ± 0.04^A^	22.42 ± 0.21^Bc^
C18:0	10.84 ± 1.11^A^	12.01 ± 0.19^Aa^	12.65 ± 0.25^A^	12.28 ± 0.12^Aa^	10.94 ± 1.53^A^	12.09 ± 0.06^Aa^
C20:0	0.51 ± 0.03^A^	0.14 ± 0.01^Bab^	0.13 ± 0.07^A^	0.16 ± 0.01^Aa^	n.d.	0.12 ± 0.01^b^
∑SFAs	38.10 ± 3.19^A^	40.40 ± 0.36^Aa^	40.84 ± 0.77^A^	36.73 ± 0.14^Bb^	38.09 ± 1.38^A^	35.44 ± 0.29^Bc^
C16:1	3.92 ± 0.19^A^	1.93 ± 0.01^Ba^	3.48 ± 0.12^A^	1.20 ± 0.03^Bb^	4.06 ± 0.44^A^	0.89 ± 0.09^Bc^
C18:1n9c	45.41 ± 2.69^A^	37.99 ± 0.07^Ba^	44.93 ± 0.55^A^	36.08 ± 0.27^Bb^	44.69 ± 1.19^A^	33.37 ± 0.37^Bc^
C20:1	0.83 ± 0.20^A^	0.56 ± 0.01^Bb^	0.94 ± 0.09^A^	0.62 ± 0.01^Ba^	0.82 ± 0.09^A^	0.42 ± 0.06^Bc^
∑MUFAs	50.16 ± 2.68^A^	40.48 ± 0.05^Ba^	49.35 ± 0.51^A^	37.89 ± 0.29^Bb^	49.57 ± 1.72^A^	34.68 ± 0.31^Bc^
C18:2n6c	10.26 ± 0.43^B^	18.40 ± 0.39^Ac^	9.43 ± 1.24^B^	24.29 ± 0.25^Ab^	11.13 ± 1.42^B^	28.80 ± 0.51^Aa^
C18:3n3 C20:2	1.03 ± 0.04^A^ 0.45 ± 0.13	0.53 ± 0.02^Bb^ n.d.	0.22 ± 0.01^B^ 0.26 ± 0.12	0.84 ± 0.01^Aa^ n.d.	0.39 ± 0.09^B^ 1.67 ± 0.01	0.81 ± 0.09^Aa^ n.d.
C20:4	n.d.	0.19 ± 0.01^b^	n.d.	0.24 ± 0.01^a^	n.d.	0.28 ± 0.03^a^
∑PUFAs	11.74 ± 0.51^B^	19.12 ± 0.40^Ac^	9.80 ± 1.28^B^	25.38 ± 0.27^Ab^	12.34 ± 0.34^B^	29.88 ± 0.56^Aa^

SFAs，Saturated Fatty Acids；MUFAs，Monounsaturated Fatty Acids；PUFAs，Polyunsaturated Fatty Acids. Different lowercase letters (abc) in the same row indicate significant differences among the three groups of sausages (*p* < 0.05), and different uppercase letters (AB) in the same row indicate significant differences between the raw meat and sausages within the same group of raw meat (*p* < 0.05).

Compared to the raw meats, the fatty acid composition of the fermented sausages changed significantly, with a significant reduction in SFAs in binary and ternary crossbred pork sausages (*p* < 0.05), while remaining unchanged in Pure Bamei pork sausages. Compared to the raw meat, the content of MUFAs significantly decreased in all three groups sausages, while the content of linoleic acid significantly increased after fermentation. The linoleic acid content in sausages of Pure Bamei pork, binary crossbred pork, and ternary crossbred pork was 1.79, 2.57, and 2.58 times higher than that of the raw meats respectively, resulting in a significant increase in the content of PUFAs in the sausages. The C20:2 fatty acid present in the raw meats was undetectable at the end of fermentation, and undetectable C20:4 in the raw meat was found in all three groups of fermented sausages.

Comparison of the fatty acid composition of sausages made from three different meat revealed significant differences in the content of SFAs, MUFA and PUFAs among the groups (*p* < 0.05). The saturated fatty acid content of 40.40 % in the sausages of Pure Bamei pork was significantly higher than those of 36.73 % and 35.44 % in the sausages of binary and ternary crossbred pork. The content of palmitic acid (C16:0) in sausages of Pure Bamei pork, binary crossbred and ternary crossbred pork was 26.97 %, 23.30 %, and 22.42 %, respectively, with significant differences observed (*p* < 0.05). Oleic acid (C18:1n9c) was the predominant MUFA, with contents of 37.99 %, 36.08 %, and 33.37 % in sausages with Pure Bamei, binary crossbred and ternary crossbred pork, respectively. The content of PUFAs significantly varied among the three groups (*p* < 0.05), with the lowest content of 19.12 % found in Pure Bamei pork sausages, compared to 29.88 % and 25.38 % in ternary crossbred and binary crossbred pork sausages, respectively. PUFAs were dominated by linoleic acid (C18:2n6c). Differences in linoleic acid content in raw meat were not significant, but the content in sausages of ternary and binary crossbred pork were 28.80 % and 24.29 %, respectively, while that in Pure Bamei pork sausages was only 18.40 %. Wang et al. also observed the increase in PUFAs in fermented sausages and attributed this to the growth and metabolism of microorganisms during fermentation (Wang et al., 2021).

### 3.5 Analysis of volatile organic compounds

**Fig. 5:**
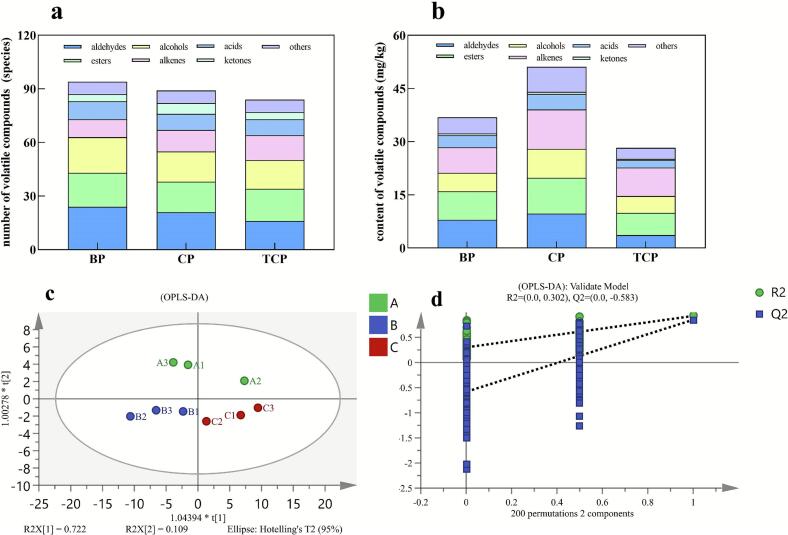
The number of volatile compounds (a), and content of volatile compounds (b) of matured sausages; The OPLS-DA model (c) and model permutation test results (d) for distinguishing the volatile flavor compounds in sausages. BP, Pure Bamei pork group; CP, binary crossbred pork group; TCP, ternary crossbred pork group.

The volatile flavor compounds of the three groups of sausages are shown in Fig. 5a and b and detailed in Supplementary Table S1, including a total of 117 volatiles. These comprise 24 aldehydes, 23 alcohols, 18 alkanes, 21 esters, 11 acids, 6 ketones, and 14 other compounds, with aldehydes, alcohols, and esters having the highest variety and content. A total of 89 volatile compounds were identified, dominated by aldehydes and esters in naturally fermented pork sausages in a study by Peng Yang (Yang et al., 2022).

To investigate the differences in volatile compounds among sausages made from different pork, a total of 64 volatile compounds common to the three groups of pork sausages were considered as the dependent variables, with the different types of raw meat as the independent variables. An OPLS-DA (Orthogonal Partial Least Squares Discriminant Analysis) model was employed to effectively discriminate the volatile profiles among the three groups of pork sausages (Fig. 5c). The independent variable fit index (Rx2) in this analysis was 0.831, and the dependent variable fit index (Ry2) was 0.775, and the model prediction index (Q2) was 0.637. Both R2 and Q2 values were greater than 0.5, indicating an acceptable model fit. After 200 permutations, the results, as shown in Fig. 5d, demonstrated that the Q2 regression line intersected the y-axis below zero, suggesting that the model was not over fitted and was thus validated. The repeatability of the volatile substances of sausages made from the same raw meat was satisfactory, while significant differences were observed between the volatile flavor compounds of sausages made from different raw pork.

The total content of aldehydes in the three groups of sausages was notably high, with the Pure Bamei pork sausages containing 24 distinct aldehyde species, which was more than those found in binary crossbred pork sausages (21 species) and ternary crossbred pork sausages (16 species), with a significant difference. Some of these aldehydes, such as citral, tridecanal, and pentadecanal, were only present in the Pure Bamei pork sausages. Rosario reported that dry-cured meat products from different types of pork showed significant differences in some aldehydes and ketones. (Ramírez & Cava, 2007). The categories of esters in the three groups of sausages were similar. Some of the esters, such as ethyl linoleate (232.99 μg/kg), ethyl palmitate (707.76 μg/kg), and ethyl acetate (142.12 μg/kg), were higher in the ternary crossbred pork sausages compared to the other two groups of sausages, Ethyl palmitate is characterized by a faint creamy aroma and ethyl acetate presents a refreshing fruity scent, both of which can provide an aromatic odour to the sausage (Sun et al., 2022). The ternary crossbred pork sausages also contained ethyl stearate, which was not detected in the other sausages. The relatively low thresholds for substances such as ethyl acetate and ethyl stearate in meat products can contribute to the special flavor of ternary pork sausages (Chang et al., 2021).

The total content of alkene compounds in the three groups of sausages was similar, but the category of alkenes was richer in the ternary crossbred pork sausage. Caryophyllene, hinokiol, thujopsene, styrene, and cedarene were only detected in the ternary crossbred pork sausages with a total of 920.2 μg/kg, all of which have distinctive aromas (Lyu et al., 2025). The threshold values for compounds such as α-pinene in meat products are 47 μg/kg, imparting a strong woody and pine-like flavor (Vespermann et al., 2017). In this experiment, all three types of pork sausages exhibited relatively high concentrations of α-pinene, with the highest level found in the binary pork sausage at 388.6 μg/kg. Meanwhile, thujanol with a minty fragrance and 2-pentylfuran with fruity scent, which were not found in the other sausages, were also detected in the ternary crossbred sausages. The extremely low thresholds of these two substances enable them to impart a distinctive flavor to the sausages (Bhatia et al., 2008). The higher sensory scores of sausages made from ternary crossbred pork may be related to these special volatile flavor compounds, such as terpene, amylfuran and ethyl acetate.

### 3.6 Analysis of bacteria community

**Fig. 6:**
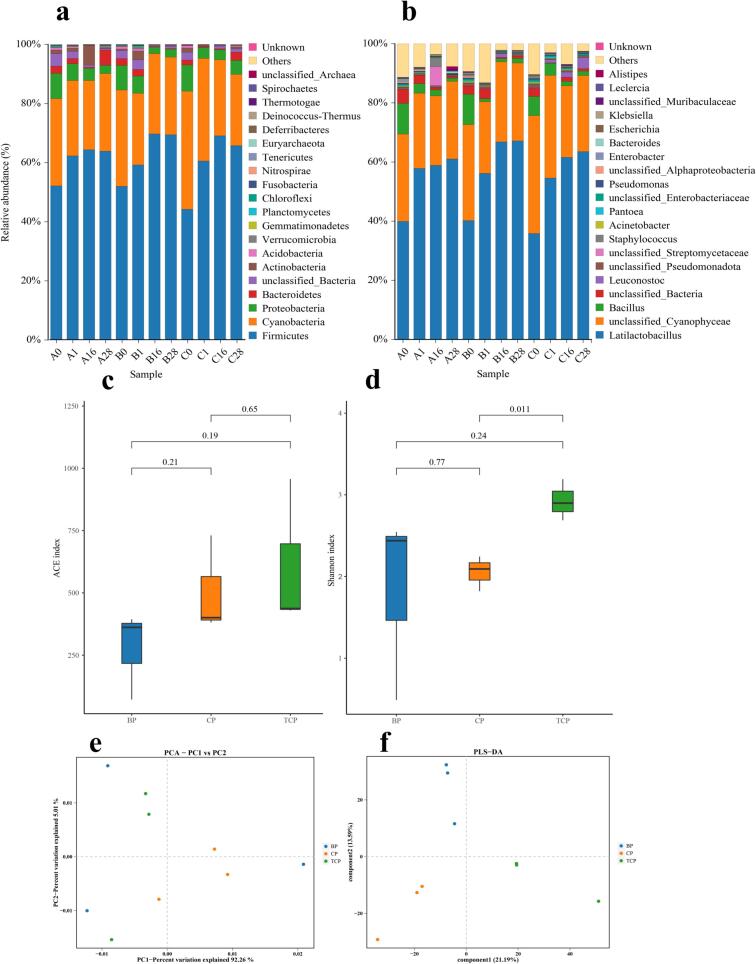
Bacterial species classification of matured sausages (a, b), as well as analysis of species abundance and diversity (c, d, e, f). A0, Pure Bamei pork Sausages on the 0th day of fermentation; B0, binary crossbred pork Sausages on the 0th day of fermentation; C0, ternary crossbred pork Sausages on the 0th day of fermentation. BP, Pure Bamei pork group; CP, binary crossbred pork group; TCP, ternary crossbred pork group.

As shown in Fig. 6a, Samples from days 0, 1, 16 and 28 were selected to analyze the bacterial community succession (Hultman et al., 2020), exhibiting the top 20 species classifications. The phylum-level bacterial classifications in sausages made from different raw meat did not differ much. The dominant bacterial phyla included Firmicutes, Cyanobacteria, Proteobacteria, and Bacteroidetes. It was Firmicutes that dominated throughout the fermentation process, displaying a trend of initial increase followed by a decrease, with the highest relative abundance at day 16, both reaching more than 60 %. Cyanobacteria was the second abundant group of bacteria in the sausages, along with Proteobacteria and Bacteroidetes, both of which declined in abundance as fermentation progressed. In the study of Katani, sausages made from edible forest game meat also predominantly consisted of Firmicutes (67.8 %), Proteobacteria (18.4 %), Cyanobacteria (8.9 %), and Bacteroidetes (3.1 %) (Katani et al., 2019).

Fig. 6b illustrates the composition at the bacterial genus level. *Lactobacillus*, *Cyanobacteria*, *Micrococcus*, *Proteus*, and *Bacillus* were the dominant genera in the sausages. *Lactobacilli* grew rapidly at the onset of fermentation, with an abundance of about 60 % after 1 day and then remained stable, a result similar to study of Wang et al. (Wang et al., 2021). The overall distribution of microbial communities at the genus level was similar in the three groups of sausages, but differed significantly in the relative abundance of certain bacteria. The relative abundance of *Micrococcus* in the matured ternary crossbred sausages was significantly higher than in the other two groups of sausages. The research by Ma et al. indicated that *Micrococcus* was also key microorganism in naturally fermented sausages (Ma et al., 2023).

The results of the bacterial α and β diversity analyses of the three groups of matured sausages are shown in Figs. 6c-f. The ACE index revealed no significant differences in species richness among the three groups of sausages. The Shannon index indicated that among the three groups of sausages, the highest bacterial species diversity was found in the ternary pork sausage, which differed significantly from the binary pork sausage (*p* < 0.05). Significant differences in the bacterial diversity of fermented sausages made from different raw meats were also found in a study by Amponsah (Amponsah et al., 2024).

The PLSDA (Partial Least Squares Discriminant Analysis) results demonstrated that the overall bacterial distribution of the three groups of sausages was distinct, allowing for their effective discrimination. Specifically, PCA (Principal Component Analysis) revealed that the bacterial community structures of the ternary and binary crossbred sausages were more similar to each other, while the Pure Bamei pork sausage exhibited a lower similarity with these two groups.

### 3.7 Analysis of non-volatile metabolites

**Fig. 7:**
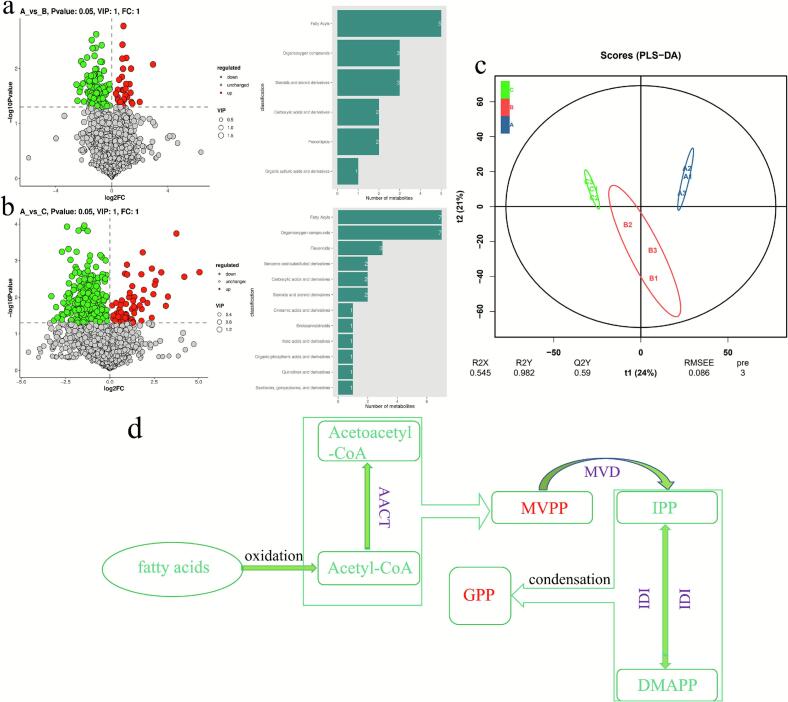
Classification of differential metabolites in 3 groups of sausages; comparison of metabolites between binary pork and Pure Baimei pork sausages (a), and between ternary pork and Pure Baimei pork sausages (b); overall differences in metabolites among the 3 groups of sausages (c); biosynthesis of the monoterpene compound precursor GPP (d).Acetyl-CoA, Acetyl coenzyme A; Acetoacetyl-CoA, Acetoacetyl coenzyme A; MVPP, phosphorylated mevalonic acid; IPP, isopentenyl pyrophosphate; DMAPP, dimethylallyl pyrophosphate; GPP, Geranyl diphosphate; AACT, acetyl-CoA *C*-acetyltransferase; MVD, mevalonate diphosphate decarboxylase; IDI, isopentenyl-diphosphate delta isomerase.

Metabolomic analysis of fermented sausages revealed a total of 2333 annotated metabolites in all samples as shown in Fig. 7. The main categories identified were acyl lipids, carboxylic acids and their derivatives, benzene and its substituted derivatives, steroids and their derivatives, and organic oxygen-containing compounds. The study of Rocchetti et al. also showed that fatty acyls and amino acids in sausages were the two most important metabolites (Rocchetti et al., 2021).

Under the parameters of a fold change (FC) ≥ 1, variable importance in projection (VIP) ≥ 1, and a significance threshold *P*-value ≤0.05, the comparative analysis of differential metabolites between binary crossbred pork and Pure Bamei pork sausages is shown in Fig. 7a. There were 111 down-regulated metabolites and 35 up-regulated metabolites, and the up-regulated metabolites contained the most variety of fatty acyls, including docosahexaenoic acid, isobutyric acid, eicosatrienoic acid, heptadecanoic acid, and arachidonic acid predominating. The comparison of metabolites between ternary crossbred pork and Pure Bamei pork sausages is shown in Fig. 7b, with 317 downregulated metabolites and 66 upregulated ones, and the largest category of up-regulated metabolites was also fatty acyls, such as hexacosadienoic acid, stearic acid, heptadecanoic acid, linoleic acid, and eicosapentaenoic acid.

The overall differences in metabolites among the three groups of sausages are shown in Fig. 7c. The metabolites of the three groups of sausages were uniformly distributed in different regions, with good repeatability of metabolites within groups and greater variability of sausages between groups. The PLS-DA model indicates a significant difference in metabolites among the three groups of sausages. The most significant metabolic differences in the fermentation of binary and ternary pork crossbred sausages in comparison with Pure Bamei pork sausage focused on lipid metabolism, which is consistent with the results of the changes in the composition of MUFAs and PUFAs in crossed pork sausages during fermentation.

KEGG pathway enrichment analyses are shown in Fig. 2S and Fig. 3S. The results further revealed that the differential metabolite pathways between binary pork and Pure Bamei pork sausages were involved in sesquiterpene biosynthesis, leucine biosynthesis, and fatty acid elongation processes. The differential metabolite pathways between ternary pork and Pure Bamei pork sausages were mainly associated with linoleic acid metabolism, sesquiterpenes and triterpenes, and unsaturated fatty acid biosynthesis. PUFAs, such as linoleic acid, which are abundant in foods, play key roles in inflammation regulation, cellular signaling, and metabolic functions (Badawy et al., 2023). Additionally, ethyl acetate, amyl furan esters, siderophores, and terpenes are important flavor compounds in sausages, significantly contributing to their flavor profile (Zhang et al., 2020).

In the comparative metabolite analysis between ternary crossbred pork and Pure Bamei pork sausages, a significant increase in the content of phosphorylated mevalonic acid (MVPP) was observed. MVPP is a crucial intermediate in the synthesis of terpene precursors isopentenyl diphosphate (IPP) and dimethylallyl diphosphate (DMAPP), which play a significant role in the biosynthesis of terpenoids (Wang et al., 2023). The elevation of MVPP content is contribute to the generation of terpenes (Bian et al., 2017). In the detection of volatile flavor compounds, specific terpenoid compounds were detected only in the ternary crosscred pork sausages with elevated MVPP content. The MVPP pathway originates from acetyl-CoA (Tetali, 2019), a central metabolite generated through fatty-acid metabolism. The terpenoid production pathway is speculated in Fig. 7d. The higher content of PUFAs in ternary crossbred pork sausages, which are easily oxidized due to their numerous unsaturated double bonds to generates acetyl-CoA, providing a foundation for the synthesis of MVPP. MVPP then synthesizes IPP and DMAPP, the common precursors for all terpenes. The condensation of these compounds can form various monoterpene precursors, which can synthesize different monoterpene compounds, such as sabinene, terpinolene, as well as cedrene, cedarene, and other sesquiterpenes.

### 3.8 The relationship between microbial communities, sensory scores, and fatty acids as well as volatile compounds

**Fig. 8:**
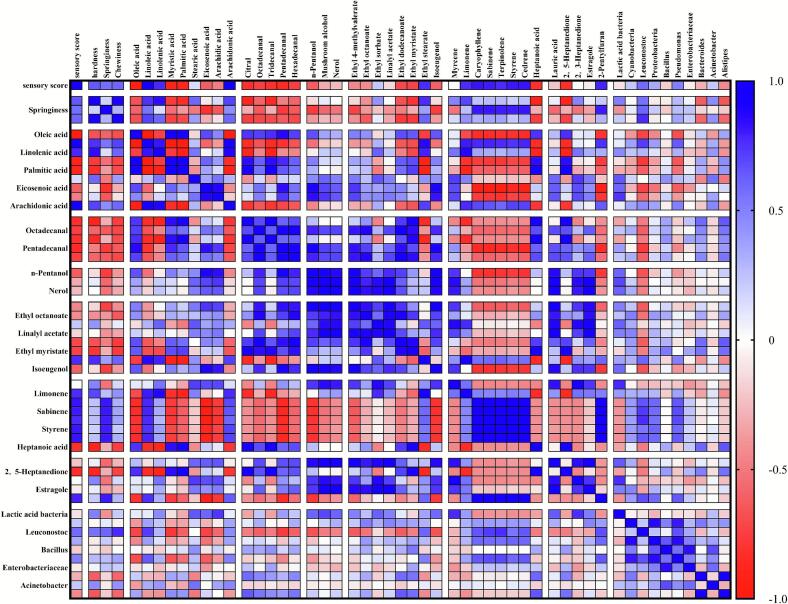
The correlation between microorganisms and sausage sensory scores, texture characteristics, fatty acids, and volatile compounds. Blue indicates a positive correlation, red indicates a negative correlation, and the deeper the color, the stronger the correlation. (For interpretation of the references to color in this figure legend, the reader is referred to the web version of this article.)

The quality and flavor of sausage is closely related to the microbial communities during sausages fermentation. The correlation between various physicochemical indexes and fatty acid composition as well as volatile compounds of the three groups of sausages is shown in Fig. 8. An OPLS-DA (Orthogonal Partial Least Squares Discriminant Analysis) model was constructed to conduct joint analyses with the top ten bacterial genera in terms of abundance and volatile flavor compounds with VIP values ≥1, fatty acids, and textural parameters.

The textural properties of sausages, including hardness, elasticity, and chewiness, exhibit a positive correlation with sensory scores. In terms of fatty acid composition, linoleic acid, linolenic acid, and arachidonic acid are positively correlated with sensory evaluation, while oleic acid and myristic acid are negatively correlated with sensory scores. Compared to pure Bamei pork sausages, the most prominent metabolic changes in crossbred pork sausages during fermentation are concentrated in lipid metabolism, with a significant increase in PUFAs content. This is one of the reasons for the marked improvement in their aroma sensory scores. Among volatile flavor compounds, aldehydes such as citral and alcohols such as n-pentanol show a negative correlation with sensory scores, as do the caprylic acid and lauric acid. Ethyl sorbate and ethyl stearate are positively correlated with sensory scores. Most of the terpene volatiles, such as cedrene were positively correlated with sensory scores, as were chrysanthenone and 2-pentylfuran. The hardness and chewiness of sausages made from ternary crossbred pork were higher, PUFAs, especially linoleic acid, was the highest among three sausages. Additionally, the content of sesquiterpenoids such as linolenic acid, arachidonic acid, and cedrene in ternary crossbred sausages is significantly increased. All of the above were positively correlated with sensory scores, endowing sausages of ternary crossbred pork with a unique flavor profile and quality.

A joint analysis of the correlations between microbes in sausages and their sensory characteristics, fatty acid composition, and volatile compounds revealed *Lactobacillus* and *Leuconostoc* as core functional genera. The relative abundance of *Lactobacillus* did not differ significantly in the three groups of matured sausages, but the relative abundance of *Leuconostoc* in the ternary crossbred pork sausages was significantly higher than that in the others. There was no significant correlation between *Lactobacillus* and the sensory quality of the sausages, while a strong positive correlation was found between *Leuconostoc* and both sensory and textural properties. The correlation between *Lactobacillus* and *Leuconostoc* with fatty acid composition was significantly different. *Lactobacillus* showed strong positive correlation with linolenic acid, stearic acid, eicosenoic acid and arachidonic acid. *Leuconostoc* showed a positive correlation with PUFAs such as linoleic acid, linolenic acid, and arachidonic acid, especially with linoleic acid. The study of Wang found that both *Lactobacillus, Pediococcus,* and *Enterococcus* were positively correlated with linoleic acid and linolenic acid, further serving as the primary sources for most volatile flavor compounds (Wang et al., 2022). Most alcohols and esters among the volatile flavor compounds were positively correlated with *Lactobacillus*. In addition, *Bacteroides* and *Prevotella* also showed positive correlation with most aldehydes, alcohols, and esters, and contributed in the synthesis of volatile flavor compounds.

## 4 Discussion

### 4.1 Variations in A_w_ and pH

The water activity (A_w_) values of sausages produced from ternary crossbred pork were significantly lower than those of Pure Bamei pork sausages (*p* < 0.05). The reduction in A_w_ values in sausages is generally attributed to decreases in pH due to bacterial growth and moisture loss (Cavalheiro et al., 2019). Given the non-significant differences in pH values among the three groups, the observed variation in A_w_ values is likely due to differential water loss from the raw meats. As shown in Table S3, the water loss rates of binary and ternary crossbred pork were much higher than that of Pure Bamei pork. In the study by Xi et al., Pure Bamei pork exhibited superior water retention, with significantly lower water loss and drip loss compared to binary and ternary crossbred pork (Xi et al., 2020). During fermentation, binary and ternary crossbred pork are more prone to moisture loss, resulting in lower A_w_ values.

### 4.2 Color and texture parameters

There was no significant difference in the color of the three raw meats as shown in Table S2. The redness values (a*) were similar and not significantly different among the groups at the end of fermentation (*p* > 0.05). However, the L* values of binary and ternary crossbred pork sausages were significantly lower than those of Pure Bamei pork sausages (*p* < 0.05), and the b* values of binary pork sausages were significantly lower. Both L* and b* values indicate the degree of sausage oxidation (Lee et al., 2024). The Pure Bamei pork sausages had the highest L* and b* values, indicating more oxidation than the crossbred pork sausages, which may be related to the fat composition of different types of pork.

The hardness and chewiness of sausages made from ternary and binary crossbred pork were significantly higher than those of Pure Bamei pork sausages, and the springiness of the ternary crossbred pork sausage was also significantly higher than that of the Pure Bamei pork sausage (*p* < 0.05). Pure Bamei pork have good water retention and high intramuscular fat content of 6.29 %, along with excellent fat deposition capabilities (Zhang et al., 2014). These differences in characteristics not only directly affect the texture of the sausage but also influence moisture changes during fermentation, thereby impacting on changes in the microbial community. The abundance of *Leuconostoc* was significantly higher in crossbred pork sausages than in purebred variants. As a heterofermentative genus, *Leuconostoc* metabolizes glucose via the phosphoketolase pathway (PKP), producing ethanol, acetate, lactate and CO₂ (Bintsis, 2018). The released carbon dioxide creates micro-pores within the sausage matrix, thereby exhibiting significantly greater springiness and earned superior sensory scores for tissue morphology relative to purebred controls. Liu et al. proposed that raw meat characteristics, microbial activity, and fermentation processes collectively contribute to textural variations in fermented sausages, where pH and water activity (A_w_) participate in texture formation through protein cross-linking (Liu et al., 2024). Future work should compare protein composition among the three pork types and its impact on fermentation to clarify the basis of their textural differences.

### 4.3 TVB-N and histamine

The production of total volatile basic nitrogen (TVB-N) is positively correlated with microbial proliferation and the activity of proteolytic enzymes which also facilitate the formation of ammonia, biogenic amines, and other products from the deamination and decarboxylation of amino acids (Sun et al., 2018). The TVB-N values were not significantly different among all groups (*p* > 0.05). However, the histamine content in sausages made from binary and ternary crossbred pork was significantly lower than that in sausages from Pure Bamei pork. Schirone et al. suggested that the main pathway for biogenic amine formation during fermentation is the microbial decarboxylation of free amino acids (Schirone et al., 2022). The bacterial community structure was found to be more similar in ternary and binary crossbred sausages compared to Pure Bamei sausages.

Histamine production was significantly lower in sausages with crossbred pork than with Pure Bamei pork, likely due to differences in microbiological activity. As shown in Table S4, the protein content of the raw material of binary and ternary crossbred pork was significantly lower than that of the Pure Bamei pork (*p* < 0.05), and it is necessary to study the amino acid composition of these different raw meats, as this also related to biogenic amine production.

### 4.4 Metabolic differential analysis

Different raw meats possess distinct muscle fiber structures, intramuscular fat content, moisture levels, pH values, and flavor profiles, which inevitably result in differential metabolism by microbial influence (Lee et al., 2018). Metabolism analysis demonstrated the major differential metabolites across the three groups as acyl lipids, carboxylic acids and their derivatives, benzene and its substituted derivatives, steroids and their derivatives, and organic oxygen-containing compounds. Compared with Pure Bamei pork sausages, the most pronounced metabolic differences in the fermentation process of binary and ternary crossbred pork sausages were focused on lipid metabolism pathways. At the end of fermentation, significant reductions in SFAs were observed in both binary and ternary crossbred pork sausages (*p* < 0.05), while PUFAs increased significantly in all three groups of sausages (*p* < 0.05). Notably, linoleic acid levels were much higher in sausages made from binary and ternary crossbred pork than sausages of Pure Bamei pork, with the most significant increase found in ternary crossbred pork sausages (*p* < 0.05). Furthermore, linoleic acid has higher nutritional value for humans as an essential fatty acid. The ternary crossbred pork sausages had a higher content of PUFAs, especially the linoleic acid, contributing better flavor and nutrition of the sausages.

KEGG pathway enrichment analysis further revealed that the metabolic differences between ternary crossbred sausages and Pure Bamei sausages were primarily concentrated in linoleic acid metabolism, sesquiterpenes and triterpenes, and the biosynthesis of unsaturated fatty acids. Metabolomics analysis revealed significant differences in lipid metabolism among the three groups of sausages. These differences led to changes in fatty acid composition during fermentation and further influenced the formation of volatile flavor compounds. Ternary crossbred sausages exhibit superior flavor characteristics due to their unique ability to generate specific terpenoids as shown in Fig. 7d. The rich content of PUFAs in Ternary crossbred sausages readily oxidizes to form acetyl-CoA to the precursor of MVPP, leading to IPP and DMAPP, universal terpene precursors. Subsequent condensation reactions yield diverse monoterpenes (e.g., sabinene, terpinolene) and sesquiterpenes (e.g., cedrene, cedarene), shaping the distinct aroma profile of ternary crossbred sausages. Ternary crossbred pork sausages are rich in PUFAs and the production of terpene compounds gives the sausage a special flavor and is the preferred raw meat for sausage production.

### 4.5 Roles of key microorganisms

The bacterial community structure of Pure Bamei pork sausages was significantly different from that of ternary and binary crossbred pork sausages, with the latter two being more similar. This suggests that differences in raw meat influence the structure and abundance of the bacterial community in sausages. *Lactobacillus* and *Leuconostoc* are key functional genera. The abundance of *Lactobacillus* was consistent across all sausage groups, while *Leuconostoc* was significantly higher in crossbred pork sausages, particularly in ternary crossbred sausages. In this study, *Lactobacillus* and *Leuconostoc* exhibited strong positive correlations with linolenic acid. Similar to the findings of Wang et al. (2022), *Lactobacillus*, *Lactococcus* and *Enterococcus* were positively correlated with linoleic and linolenic acids. Wang et al. demonstrated that *Leuconostoc* is positively correlated with the metabolism of PUFAs, which are closely linked to flavor compounds in fermented sausages (Wang et al., 2022). The differences in linoleic acid content among the raw meats were not significant (*p* > 0.05). The linoleic acid content in sausages made from crossbred pork was significantly increased, especially in ternary crossbred pork, which showed a 56.5 % increase compared to that in Pure Bamei pork. Linoleic acid is a precursor to numerous flavor compounds. In a study by Zhou et al., it was found that the degradation of linoleic acid alone or in reaction with other substances during heating of meat products resulted in the formation of a very large variety of volatile (Zhou et al., 2022).

Additionally, n-pentanol was positively correlated with *Lactobacillus*. Citral, tridecanal and pentadecanal was positively correlated with *Bacillus and Enterobacteriaceae.* Styrene, and cedarene positively correlated with *Leuconostoc* and *Enterobacteriaceae.* Ethyl sorbate and ethyl acetate was positively correlated with *Lactobacillus* but negatively with *Leuconostoc.* The low-threshold compound 2-pentylfuran was positively correlated with *Leuconostoc*, contributing to the rich flavor profile of ternary crossbred pork sausages consistenting with high levels in ternary sausages. Studies have shown that *Leuconostoc* can participate in the incomplete oxidation of fatty acids, primarily producing acids and ketones through its metabolic activities, which further serve as precursors for ester formation (Tang et al., 2019). Remarkably, *Leuconostoc* exhibited a significant and robust correlation with the majority of terpenoid volatiles in the present study. Based on metabolomic evidence, we hypothesize that *Leuconostoc* is involved in a metabolic pathway wherein PUFAs are oxidized to form the MVPP. This precursor is subsequently utilized for the synthesis of the universal terpenoid precursors, isopentenyl pyrophosphate IPP and dimethylallyl pyrophosphate DMAPP. These findings demonstrate that flavor development depends critically on fat decomposition and oxidation. This process is intensified in crossbred pork, particularly ternary crossbred, by fostering a functional microbial consortium of *Lactobacillus* and *Leuconostoc*, which enhances fatty-acid metabolism and ultimately delivering a more complex flavor profile in the fermented sausages.

## 5 Conclusion

Differences of Chinese sausages produced from Pure Bamei Pigs and its binary crossbred and ternary crossbred pork were evaluated. The three different raw meat had no effect on pH changes during sausage fermentation. The A_w_ values of sausages made from both binary and ternary pigs were significantly lower than those from Pure Bamei pork. The highest overall acceptability scores were obtained in the sausages from the ternary crossbred pork. The linoleic acid in the binary and ternary pork sausages was significantly higher than that in the sausages of Pure Bamei pork. The content of esters such as ethyl linoleate and ethyl palmitate were higher in the ternary pork sausages, and the olefin categories such as caryophyllene and hinokiene were richer, which conferred a unique flavor to the sausages. Bacterial community structure analysis showed no significant difference in species abundance values among the three groups of sausages, but higher species diversity was found in the ternary pork sausages. The most important metabolic differences among the three groups of sausages were mainly focused on lipid metabolism, and the main differentiators were fatty acyl substances and oxygenated organic substances. *Lactobacillus* and *Leuconostoc* were the core functional genera. Ternary crossbred pork was identified as the preferred raw material for making fermented sausages. Future research will focus on how the physicochemical properties of raw meat influence the mechanisms of flavor compounds production by shaping the core microbial community and regulating key enzymatic pathways.

## CRediT authorship contribution statement

**Sen Xiang:** Writing – original draft, Investigation, Data curation. **Youqing Wang:** Investigation, Formal analysis. **Jiale Zheng:** Software, Investigation. **Zhongfang Tan:** Resources, Funding acquisition. **Guofang Wu:** Supervision, Resources. **Lei Wang:** Resources. **Jianbo Zhang:** Methodology. **Xuan Luo:** Methodology. **Xin Zhang:** Project administration, Formal analysis. **Haiying Wang:** Writing – review & editing, Conceptualization.

## Informed consent statement

All respondents were assured that their participation in the survey was voluntary, and their informed consent to join or not was respected.

## Declaration of competing interest

The authors declare that they have no known competing financial interests or personal relationships that could have appeared to influence the work reported in this paper.

## Data Availability

Data will be made available on request.

## Appendix A Supplementary data

Supplementary material Image 1
