# Supplementary material for: Comparative analysis of fermented sausages from purebred and crossbred Bamei pig: physicochemical properties, fatty acids, microbiota, and metabolites
Source: Food Chem X. 2025 Nov 19;32:103299. doi: 10.1016/j.fochx.2025.103299 (PMC12951228; doi:10.1016/j.fochx.2025.103299)
Supplement: Supplementary file 1 — Supplementary material [file mmc1.docx]

**Table S1** The content of volatile substances in sausages (μg/kg)

| Types | Bamei pork | binary crossbred pork | ternary crossbred pork |
| --- | --- | --- | --- |
| Aldehydes  Valeraldehyde  Hexanal  Heptanal  2-Heptenal  Nonanal  (E,E)-2,4-Heptadienal  Benzaldehyde  (Z)-2-Decenal  (E,E)-2,4-Nonadienal  cis-Citral  Octadecanal  cis-Cinnamaldehyde  trans-2-Hexenal  1-Methylcyclohex-3-ene-1-carboxaldehyde  trans-2-Octenal  (Z)-2-Nonenal  (E,E)-2,4-Dodecadienal  trans-2,4-Decadienal  Tridecanal  α-Ethylidene-Benzeneacetaldehyde  4,5-Epoxy-(Z)-2-Decenal  Pentadecanal  Hexadecanal  cis-13-Octadecen-1-al  Total  Alcohols  4-Amino-1-pentanol  Ethanol  n-Pentanol  3-Propyl-2,4-pentadien-1-ol  4,4-Dimethylcyclohexene alcohol  Muscone  Cyclohexanol, 1-methyl-4-, cis-  trans-4-(1-Methylcyclohex-2-en-1-yl)-1-butanol  Linalool  4-Terpineol  4-Methyl-1-bicyclo[3.1.0]hexane-3-ol  trans-2-Octenol  α-Asarone  α-Terpineol  Nerolidol  Trans-Carveol  cis-P-Methane-1(7),8-dien-2-ol  Benzyl Alcohol  Phenethyl Alcohol  2-[(Z)-9-Octadecenoxyl]ethanol  Oleanol  3-Phenylprop-2-en-1-ol  Thujanol  Total  Alkenes  β-Pinene  (R)-1-Methyl-5-Cyclohexene  2-Methyl-1-Propene  -α-Pinene  (-)-Isopulegol  1,3-Cyclohexadiene, 5-2-Methyl  β-Caryophyllene  Β-Humulene  α-Curcumene  Anethole  Myrcene  Apiole  2-Methyl-1-Pentadecene  Caryophyllene  Hinokitiol  Isopulegol  Styrene  Cedrene  Total  Esters  Ethyl Butyrate  Ethyl Hexanoate  L(-)-Lactic Acid Ethyl Ester  Ethyl Octanoate  Ethyl Sorbate  Linalyl Acetate  Ethyl Dodecanoate  Ethyl Vinyl Hexoate  Terpinyl Acetate  (R)-Lavandulyl Acetate  Neryl Acetate  Ethyl Myristate  9-Oxo Nonanoic Acid Ethyl Ester  Trans-Cinnamic Acid Ethyl Ester  δ-Tetradecalactone  Ethyl Palmitate  Ethyl Acetate  Ethyl Linoleate  Butyl Phthalate  Isobutyl Octadecanoate  Ethyl Stearate  Total  Acids  Acetic Acid  Butyric Acid  Hexanoic Acid  Heptanoic Acid  Octanoic Acid  Palmitic Acid  Nonanoic Acid  Decanoic Acid  Lauric Acid  Myristic Acid  Oxirane Carboxylic Acid  Total  Ketones  3-Hydroxy-2-Butanone  2,5-Hexanedione  3,5-Octadien-2-One  3,5-Dihydroxy-2-Methyl-4H-Pyran-4-One  2,3-Octanedione  Chicory Ketone  Total  Others  2-Pentylfuran  Artemisia Ketone  Nitrosomethylurea  Tetradecane  2,6,10-Trimethyltetradecane  Pentadecane  Hexadecane  1,2-Epoxytetradecane  Chrysanthrene  Total | 0.25±0.1  893.18±466.36  85.82±31.24  419.4±159.46  389.26±129.79  91.42±34.38  737.83±276.78  565.64±209.22  302.61±121.36  13.43±5.34  1024.37±406.6  203.83±81.17  26.72±9  79.21±28.12  511.12±171.73  503.08±176.05  82.09±31.11  624.26±176.42  27.55±9.79  46.19±22.32  69.3±27.6  190.27±64.74  1024.37±406.6  16.95±8.13  7928.17±2902.49^ab^  52.69±20.97  1938.14±560.2  128.81±52.85  18.95±7.81  85.09±34.75  456.07±163.73  133.65±49.64  93.46±38.63  1202.08±436.03  300.71±110.66  38.55±15.89  83.02±31.17  101.79±41.58  289.67±113.47  26.09±10.83  19.04±7.38  16.97±6.6  105.93±52.8  80.56±32.88  24.09±9.27  n.d.  n.d.  n.d.  5196.59±1758.62^a^  147.82±62.26  503.4±182.22  6.99±3.1  264.98±101.52  147±55.8  457.15±193.31  452.08±198.08  891.8±358.98  1380.15±583.45  2971.06±1322.02  n.d.  n.d.  n.d.  n.d.  n.d.  n.d.  n.d.  n.d.  7222.44±3025.99^a^  62.74±31.25  381.25±144.42  307.43±119.18  288.03±103.54  88.59±32.56  3508.59±1366.93  1134.86±483.45  191.65±78.26  562.25±243.38  63.41±24.13  112.91±46.19  246.28±111.36  48.08±18.82  69.26±31.46  25.14±11.93  643.11±303.79  149.39±62.92  160.85±66.82  9.99±6.32  n.d.  n.d.  8060.71±3221.93^a^  725.33±377.83  243.3±101.64  860.23±372.77  30.22±11.7  198.94±74.92  5.12±2.39  475.08±139.42  498.87±214.96  112.07±41.13  229.26±90.22  n.d.  3378.42±1355.35^ab^  58.85±23.69  42.68±15.27  133.24±60.07  284.3±120.44  n.d.  n.d.  519.06±215.48^a^  n.d.  2278.95±921.35  1323.24±1162.48  242.82±98.21  27.23±8.26  385.67±150.39  132.89±51.51  138.54±48.34  n.d.  4529.34±1871.47^ab^ | 0.34±0.09  1213.68±85.37  101.43±16.2  750.23±140.47  484.29±85.43  188.3±42.71  722.08±134.74  761.45±152.27  471.01±92.51  n.d.  900.65±134.85  378.06±101.27  39±11.32  129.11±21.33  710.33±110.04  656.27±126.41  132.56±28.11  992.33±194.48  n.d.  n.d.  n.d.  175.32±15.08  900.65±134.85  18.11±3.78  9725.21±1252.69^a^  43.93±16.1  3352.12±727.61  197.07±38.47  n.d.  n.d.  764.74±124.95  203.57±34.52  n.d.  1869.23±385.18  435.49±92.07  68.85±17.91  142±31.06  147.39±30.63  430.7±93.17  42.53±7.9  n.d.  29.33±5.66  157.34±31.33  118.87±22.02  n.d.  20.68±2.8  128.56±14.54  n.d.  8152.4±1670.13^a^  36.94±5  n.d.  11.47±3.19  388.57±81.56  228.43±52.61  780.52±172.18  690.45±146.01  1406.93±288.77  2088.21±443.35  4381.02±980.56  252±52.18  768.26±119.98  94.32±28.7  n.d.  n.d.  n.d.  n.d.  n.d.  11127.12±2354.27^a^  73.74±27.33  542.44±147.04  510.51±112.05  298.52±56.08  233.51±55.45  5059.19±1072.91  904.54±211.27  379.37±69.36  624.47±156.34  149.57±36.04  n.d.  124.37±21.42  75.91±11.44  81.03±16.94  n.d.  731.14±183.47  111.13±10.14  159.63±22.92  n.d.  21.35±8.69  n.d.  10080.42±2173.55^a^  899.65±285.99  258.5±65.08  1101.4±201.14  n.d.  233.42±42.09  7.15±1.25  1122.61±128.57  402.75±81.11  181.11±30.73  177.52±23.8  n.d.  4384.12±750.19^a^  58.65±18.71  n.d.  254.88±62.1  130.75±60.07  41.53±9.96  115.09±29.68  600.91±163.06^a^  n.d.  3580.3±834.96  198.63±33.05  885.6±235.18  157.15±32.69  1487.01±424.16  543.51±181.18  180.11±40.08  n.d.  7032.3±1769.02^a^ | 0.26±0.08  666.43±311.72  37.49±14.38  192.73±79.43  231.6±79.58  48.14±24.01  342.18±137.91  213.87±80.55  108.99±45.9  n.d.  462.97±168.38  126.71±48.86  n.d.  n.d.  211.71±83.02  223.86±88.62  n.d.  283.91±71.04  n.d.  n.d.  n.d.  n.d.  462.97±168.38  21.16±9.46  3634.99±1338.53^b^  n.d.  2043.73±924.7  n.d.  n.d.  n.d.  294.29±114.89  140.86±61.66  n.d.  1246.92±518.46  299.71±122.44  35.03±15.01  58.62±21.33  82.34±52.55  287.14±118.74  18.37±8.28  16.26±6.98  n.d.  95.2±30.13  76.27±31.82  69.86±31.17  13.53±7.41  n.d.  0.19±0.12  4778.3±2053.53^a^  200.22±98.52  n.d.  4.57±2.68  251.49±106.95  n.d.  754.22±358.76  406.82±171.46  790.4±339.65  1230.83±486.63  2765.37±1067.98  n.d.  680.78±299.96  n.d.  141.9±60.03  39.3±15.17  15.21±6.9  298±165.52  425.79±175.27  8004.9±3344.09^a^  36.08±29.83  167.17±73.69  318.4±145.07  203.16±85.4  125.75±52.67  3014.16±1182.14  506.61±212.79  41.58±14.13  418.15±157.04  125.51±50.88  n.d.  104.43±40.47  33.1±10.34  53.25±22.2  n.d.  707.76±347.55  142.12±51.98  232.99±84.52  15.66±6.16  n.d.  21.96±8.34  6267.81±2550.25^a^  359.08±174.87  106.41±49.69  395.04±154.17  n.d.  114.08±41.25  n.d.  664.87±375.82  277.87±122.86  86.73±29.78  90.56±50.21  0.26±0.08  2094.9±736.31^b^  27.22±10.49  n.d.  91.28±36.56  117.2±76.57  n.d.  93.6±42.53  329.3±165.28^a^  3.42±1.75  2110.6±848.04  n.d.  268.83±114.76  38.49±17.19  450.59±169.38  198.91±71.03  n.d.  59.31±25.1  3130.15±1240.68^b^ |

**Table S2** The standard and score of sensory characteristics of sausages

| Items | Standard for evaluation | Scores |
| --- | --- | --- |
| **Appearance(20)** | Mildew stains, wet surface, sticky substance | 1-5 |
|  | Fewer surface mold spots, slightly wet and sticky surface | 6-10 |
|  | No mold and stickiness on the surface, dry enteric coating, but low elasticity | 11-15 |
|  | No mold on the surface, no stickiness, dry and elastic sausage casings | 16-20 |
| **Tissue morphology(20)** | The cut surface is rough, with obvious cracks, and the meat is loose and cannot be sliced | 1-5 |
|  | Slightly rough and cracked on the cut surface, but can be sliced | 6-10 |
|  | Delicate cuts, firmer meat, smoother and flatter cuts | 11-15 |
|  | Delicate cuts, firm meat production, smooth and flat cuts | 16-20 |
| **Color(20)** | Grayish in cross-section with poor color uniformity | 1-5 |
|  | Light reddish color in cross-section, average color uniformity | 6-10 |
|  | Reddish in cross-section, average color uniformity | 11-15 |
|  | Attractive red cross-section, good color uniformity | 16-20 |
| **Smell(20)** | No meat flavor, with a distinctly rancid odor | 1-5 |
|  | Meat flavor is not obvious, the smell has a mildly rancid taste | 6-10 |
|  | Meat flavor is not obvious, odor without rancid taste | 11-15 |
|  | Characteristic fermented flavor of fermented sausage | 16-20 |
| **Flavor(20)** | Too salty or too bland, meat falls apart and chews poorly | 1-5 |
|  | Slightly salty or slightly bland, meat slightly loose, less chewy | 6-10 |
|  | Medium saltiness, meat has some firmness and elasticity, good chewiness | 11-15 |
|  | Good flavor, firm and elastic meat, good chewiness | 16-20 |

**Table S3** The meat quality of the three types of raw meat

|  | NShear stress (kgf)/N | Loss of water rate (%) | Cooking percentage (%) | Meat color |
| --- | --- | --- | --- | --- |
| Bamei pork | 48.89±14.59^a^ | 4.05±0.37^b^ | 65.38±1.60^a^ | 4.00±0.00^a^ |
| binary crossbred pork | 35.82±7.91^b^ | 18.10±4.92^a^ | 63.43±0.36^a^ | 3.67±0.58^a^ |
| ternary crossbred pork | 38.32±7.31^b^ | 18.89±10.40^a^ | 64.63±0.80^a^ | 4.00±0.00^a^ |

In the table, different lowercase English letters indicate significant differences (P < 0.05), while the presence of the same letter indicates no significant difference (P > 0.05). The same applies below.

**Table S4** Analysis of Nutrient Content in Meat

|  | Moisture (%) | Protein (%) | Fat (%) | Ash (%) |
| --- | --- | --- | --- | --- |
| Bamei pork | 69.38±1.72^a^ | 21.19±1.14^a^ | 9.46±2.46^a^ | 1.13±0.06^a^ |
| binary crossbred pork | 69.02±1.85^a^ | 20.76±1.74^ab^ | 10.23±3.13^a^ | 1.17±0.05^a^ |
| ternary crossbred pork | 72.20±0.86^a^ | 19.46±0.33^b^ | 7.90±0.23^b^ | 1.30±0.07^a^ |


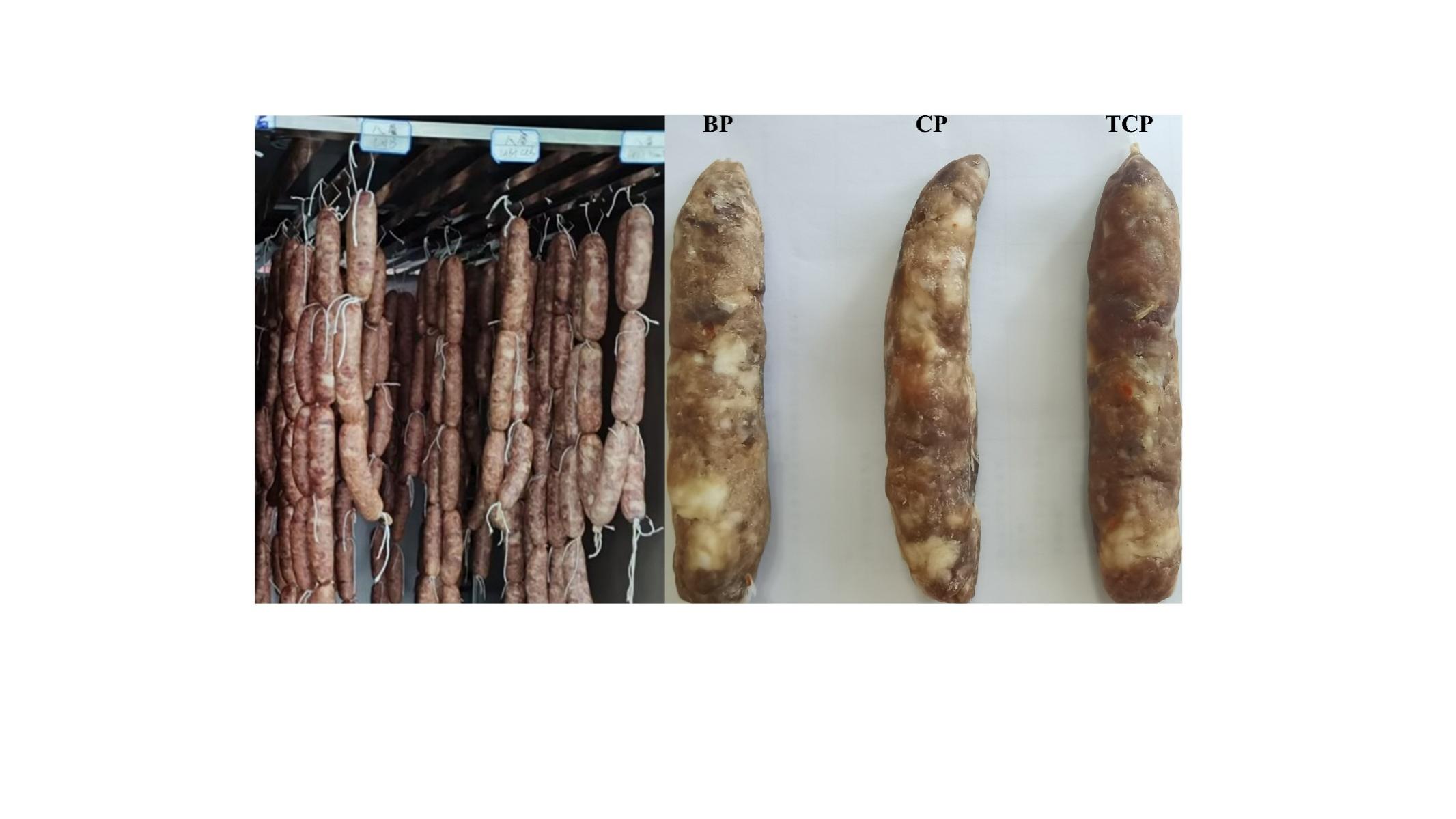


**Fig.S1** Fermented and final matured sausages. BP, Bamei pork group; CP, binary crossbred pork group;TCP, ternary crossbred pork group .


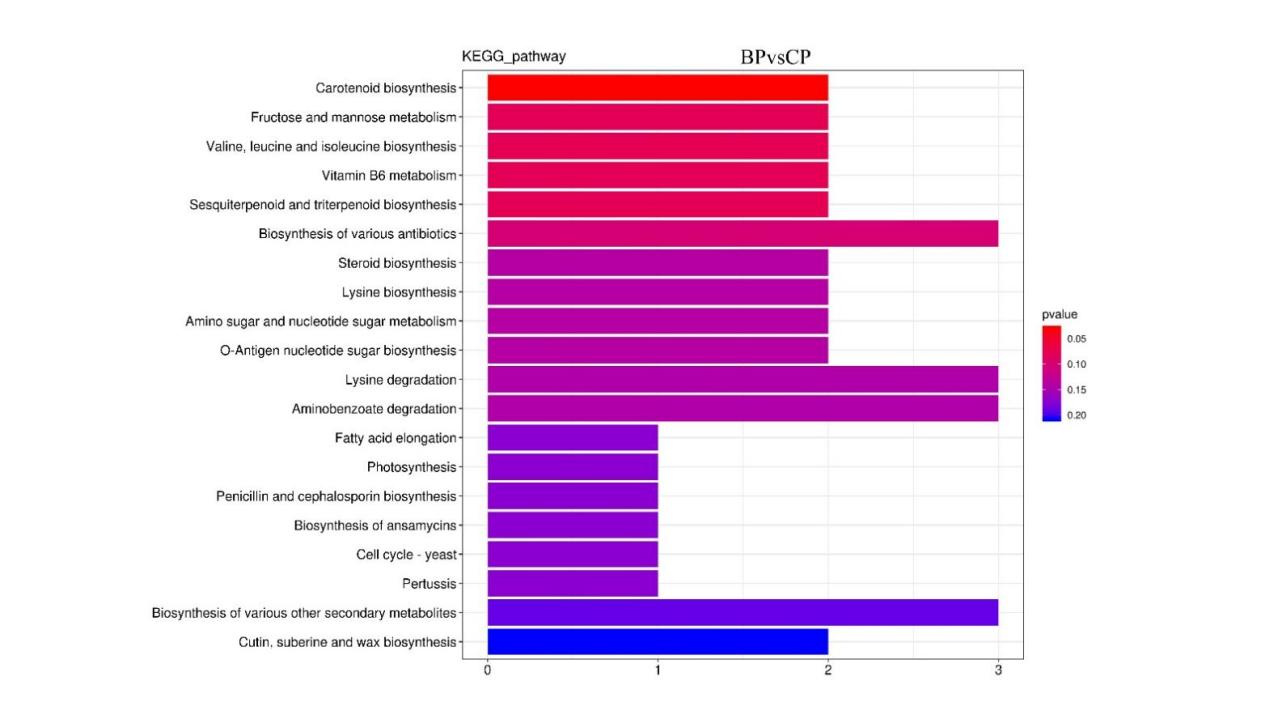


**Fig.S2** KEGG pathway enrichment analysis of differential metabolites between

Bamei pork sausage and binary crossbred pork sausage.

The x-axis represents the number of differential metabolites annotated to that pathway,

and the y-axis represents the pathway names.


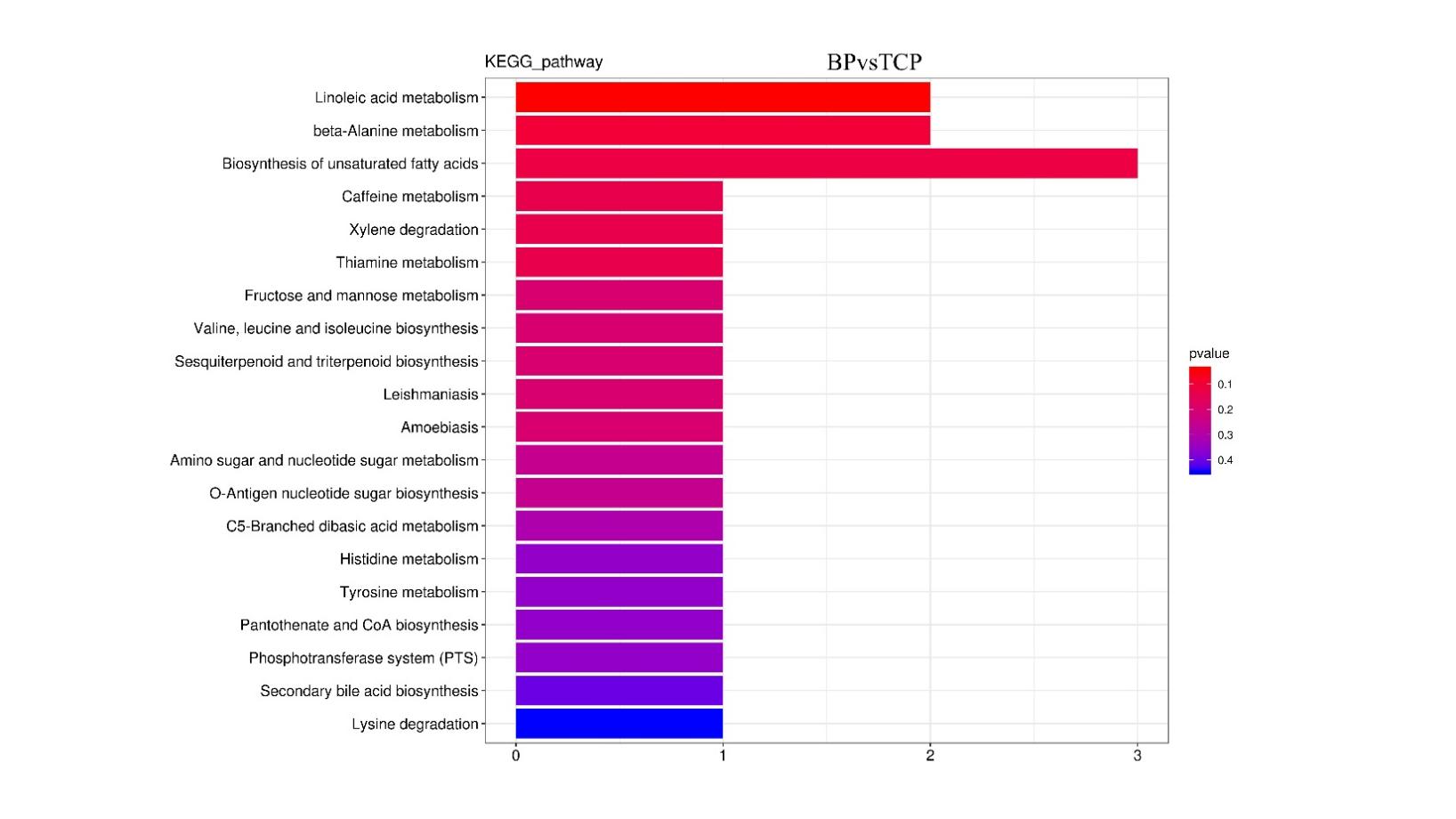


**Fig.S3** The KEGG pathway enrichment analysis of differential metabolites Bamei pork

and ternary crossbred pork sausages.

The x-axis represents the number of differential metabolites annotated to that pathway,

and the y-axis represents the pathway names.
